# Melatonin Prevents Transforming Growth Factor-β1-Stimulated Transdifferentiation of Renal Interstitial Fibroblasts to Myofibroblasts by Suppressing Reactive Oxygen Species-Dependent Mechanisms

**DOI:** 10.3390/antiox9010039

**Published:** 2020-01-01

**Authors:** Jung-Yeon Kim, Jae-Hyung Park, Eon Ju Jeon, Jaechan Leem, Kwan-Kyu Park

**Affiliations:** 1 Department of Immunology, School of Medicine, Catholic University of Daegu, Daegu 42472, Korea; jy1118@cu.ac.kr; 2 Department of Physiology, School of Medicine, Keimyung University, Daegu 42601, Korea; physiopark@kmu.ac.kr; 3 Department of Internal Medicine, School of Medicine, Catholic University of Daegu, Daegu 42472, Korea; 4 Department of Pathology, School of Medicine, Catholic University of Daegu, Daegu 42472, Korea; kkpark@cu.ac.kr

**Keywords:** transforming growth factor-β1, fibroblast-myofibroblast transdifferentiation, reactive oxygen species, renal interstitial fibroblasts, melatonin

## Abstract

Accumulating evidence suggests that the pineal hormone melatonin displays protective effects against renal fibrosis, but the mechanisms remain poorly understood. Here, we investigate the effect of the pineal hormone on transdifferentiation of renal fibroblasts to myofibroblasts invoked by transforming growth factor-β1 (TGF-β1). Increased proliferation and activation of renal interstitial fibroblasts after TGF-β1 treatment were attenuated by melatonin pretreatment. Mechanistically, melatonin suppressed Smad2/3 phosphorylation and nuclear co-localization of their phosphorylated forms and Smad4 after TGF-β1 stimulation. In addition, increased phosphorylations of Akt, extracellular signal-regulated kinase 1/2, and p38 after TGF-β1 treatment were also suppressed by the hormone. These effects of melatonin were not affected by pharmacological and genetic inhibition of its membrane receptors. Furthermore, melatonin significantly reversed an increase of intracellular reactive oxygen species (ROS) and malondialdehyde levels, and a decrease of the reduced glutathione/oxidized glutathione ratio after TGF-β1 treatment. Finally, TGF-β1-induced proliferation and activation were also suppressed by N-acetylcysteine. Altogether, these findings suggest that the pineal hormone melatonin prevents TGF-β1-induced transdifferentiation of renal interstitial fibroblasts to myofibroblasts via inhibition of Smad and non-Smad signaling cadcades by inhibiting ROS-mediated mechanisms in its receptor-independent manner.

## 1. Introduction

Renal fibrosis is critically involved in the pathogenesis of chronic kidney disease (CKD) and attributed to excessive deposition of extracellular matrix (ECM). Its underlying mechanisms are complex and involve various cellular pathways [1]. Among them, fibroblast-myofibroblast transdifferentiation is one of the critical steps in the fibrotic process. Myofibroblasts synthesize and secrete large amount of ECM into the renal interstitial region. Thus, blocking fibroblast-myofibroblast transdifferentiation would be a promising preventive or therapeutic approach against renal fibrosis.

Transforming growth factor-β1 (TGF-β1) is a crucial mediator in the pathophysiology of fibrotic diseases such as renal fibrosis [2,3]. Its downstream signaling involves Smad and non-Smad signaling cascades that regulate gene expression required for fibrotic processes including fibroblast-myofibroblast transdifferentiation. It has been shown that Smad proteins are overactivated in the kidneys of patients and animals with CKD [4,5,6,7]. TGF-β1 induces phosphorylation of Smad proteins including Smad2 and Smad3, which form complexes with Smad4 [2]. Then, the complexes are translocated into the nucleus to modulate expression of fibrosis-related genes. In addition, the cytokine can also activate various non-Smad signaling pathways such as Akt and mitogen-activated protein kinase (MAPK) pathways [8].

The pineal hormone melatonin plays an essential role in regulating the sleep–wake cycle [9]. Besides, the hormone has been reported to exert multiple biological actions such as anti-inflammatory and anti-oxidant effects [10,11]. Accumulating evidence suggests that fibrotic processes in a variety of organs were ameliorated by the hormone [12]. Indeed, melatonin was shown to have protective effects against renal fibrosis in several animal models [13,14,15,16]. However, mechanisms for the beneficial effects of the hormone against renal fibrosis remain poorly understood.

The present study aimed to explore the effects of melatonin on TGF-β1-stimulated fibroblast-myofibroblast transdifferentiation and investigate its underlying mechanisms. We noted that melatonin prevents TGF-β1-induced proliferation and activation of renal fibroblasts through suppressing Smad and non-Smad signaling cascades. These effects of melatonin were mediated by inhibiting reactive oxygen species (ROS)-mediated mechanisms in its receptor-independent manner. These findings provide a novel mechanistic insight into the preventive effects of melatonin against renal fibrosis.

## 2. Materials and Methods

### 2.1. Cell Culture and Treatments

The rat kidney interstitial fibroblast cell line NRK-49F cells were purchased from the American Type Culture Collection (Rockville, MD, USA). Cells were grown in Dulbecco’s Modified Eagle’s Medium containing 10% fetal bovine serum at 37 °C under 5% CO₂ and 95% air. To explore the effect of melatonin on TGF-β1-stimulated activation of fibroblasts, the cells were incubated with TGF-β1 (5 ng/mL; R&D Systems, Minneapolis, MN, USA) for 24 h after pretreatment with melatonin (0.1 mM or 1 mM) for 30 min in the presence or absence of luzindole (20 μM or 100 μM). In addition, the cells were treated with TGF-β1 (5 ng/mL) for 24 h after preincubation with *N*-acetylcysteine (NAC, 10 mM) or 1 mM melatonin for 30 min. Melatonin, lunzindole, and NAC were purchased from Sigma-Aldrich (St. Louis, MO, USA). Melatonin and luzindole were dissolved in dimethyl sulfoxide (DMSO). The solvent was added to the control cells in the experiments with these compounds. The final concentrations of DMSO in each well did not exceed 0.5% (*v/v*), which by itself did not affect the cell viability.

### 2.2. Cell Viability Assay

To evaluate the effect of melatonin on TGF-β1-stimulated proliferation of fibroblasts, NRK-49F cells were treated with TGF-β1 (5 ng/mL) after preincubation with melatonin (0.01 mM, 0.1 mM, or 1 mM) for 30 min in the presence or absence of luzindole (100 μM). In another experiment, cells were treated with TGF-β1 (5 ng/mL) after preincubation with NAC (10 mM) or melatonin (1 mM) for 30 min. Cell viability was analyzed using the Cell Counting Kit-8 (CCK-8; Dojindo Laboratories, Kumamoto, Japan) assay at 0, 24, and 48 h after TGF-β1 stimulation according to the manufacturer’s instructions. The absorbance at 450 nm was assessed using a microplate reader (Thermo Fisher Scientific, Waltham, MA, USA).

### 2.3. Western Blot Analysis

Western blotting was performed as described previously [17]. Briefly, protein samples were resolved by sodium dodecyl sulfate polyacrylamide gel electrophoresis (SDS-PAGE) and then transferred from the gels onto nitrocellulose membranes. The membranes were proved with primary antibodies overnight at 4 °C, followed by incubation with a horseradish peroxidase-conjugated secondary antibody for 1 h at room temperature. The following primary antibodies were used in this study: anti-collagen Ⅰ (1:1000; ab34710; Abcam, Cambridge, MA, USA), anti-fibronectin (1:1000; ab2413; Abcam), anti-α-smooth muscle actin (α-SMA; 1:1000; A2547; Sigma-Aldrich), anti-p-Smad2/3 (1:1000; #8828; Cell Signaling, Danvers, MA, USA), anti-Smad2/3 (1:1000; #3102; Cell Signaling), anti-p-Akt (1:1000; #9271; Cell Signaling), anti-Akt (1:1000; #9272; Cell Signaling), anti-p-extracellular signal-regulated kinase 1/2 (p-ERK1/2; 1:1000; #4370; Cell Signaling), anti-ERK1/2 (1:1000; #9102; Cell Signaling), anti-p-p38 (1:1000; #9215; Cell Signaling), anti-p38 (1:1000; #8690; Cell Signaling), anti-melatonin receptor type 1A (MT1; 1:1000; orb11085; Biorbyt, San Francisco, CA, USA), anti-melatonin receptor type 1B (MT2; 1:1000; NLS932; Novus Biologicals, Littleton, CO, USA), and anti-glyceraldehyde-3-phosphate dehydrogenase (GAPDH; 1:3000; #2118; Cell Signaling) antibody. The protein expression levels were normalized with GAPDH. Quantitative analysis of protein levels was performed using NIH ImageJ software (National Institutes of Health, Bethesda, MD, USA).

### 2.4. Reverse Transcription-Polymerase Chain Reaction (RT-PCR)

Total RNA was extracted from cells with TRIzol (Invitrogen, Carlsbad, CA, USA) and cDNA was synthesized from 2 ug of total RNA using RNA to cDNA EcoDry Premix (TaKaRa, Tokyo, Japan) according to the manufacturer’s instructions. The cDNA was amplified by PCR with the following primers: MT1 (NM_008639.3), 5′-TGTCAGCGAGCTGCTCAATG-3′ and 5′-GGTACACAGACAGGATGACCA-3′; MT2 (NM_145712.2), 5′-GAACAGCTCAATCCCTAACTGC-3′ and 5′-ACGACTACTGTAGATAGCATGGG-3′, GAPDH (NM_008084.2), 5′-GACAACTTTGGCATCGTGGA-3′ and 5′–ATGCAGGGATGATGTTCTGG-3′.

### 2.5. Knockdown of MT1 and MT2

NRK-49F cells were seeded onto a 60-mm culture dish and transfected with rat MT1/MT2 small interfering RNA (siRNA) (#1330001; Thermo Fisher Scientific) or control siRNA (AM4611; Invitrogen) using Lipofectamine 2000 (Thermo Fisher Scientific) according to the manufacturer’s instructions. At 24 h after transfection, the cells were pretreated with melatonin (1 mM) for 30 min and then incubated with TGF-β1 (5 ng/mL).

### 2.6. Evaluation of Iintracellular ROS and Redox Status

Amounts of intracellular ROS and malondialdehyde (MDA) were measured using the 2’,7’-dichlorofluorescin diacetate (DCFDA)-Cellular ROS Assay Kit (ab113851; Abcam) and the Lipid Peroxidation (MDA) Assay Kit (MAK085; Sigma-Aldrich), respectively, according to the manufacturer’s instructions. The reduced glutathione/oxidized glutathione ratio (GSH/GSSG) were measured using the Glutathione (GSSG/GSH) Detection Kit (ADI-900-160; Enzo Life Sciences, Farmingdale, NY, USA) according to the manufacturer’s instructions.

### 2.7. Immunofluorescence Analysis

NRK-49F cells were fixed for 20 min at room temperature with 4% paraformaldehyde in phosphate-buffered saline. After permeabilization and blocking, the cells were probed with primary antibodies against p-Smad2/3 (1:200; #8828; Cell Signaling), Smad4 (1:200; sc7966; Santa Cruz Biotechnology, Santa Cruz, CA, USA), or α-SMA (1:200; A2547; Sigma-Aldrich), followed by incubation with secondary antibodies directed against the primary antibody. Nuclei were counterstained with 4′,6-diamidino-2-phenylindole (DAPI). The stained cells were visualized using a confocal microscope (Nikon, Tokyo, Japan).

### 2.8. Statistical Analysis

Data are presented as the mean ± standard error of the mean (SEM). Comparisons between groups were assessed using one-way ANOVA with Bonferroni’s post-hoc tests. All experiments were performed at least 2 times. A *p* value less than 0.05 was considered statistically significant.

## 3. Results

### 3.1. Melatonin Inhibits TGF-β1-Induced Proliferation and Activation in NRK-49F Cells

**Figure 1:**
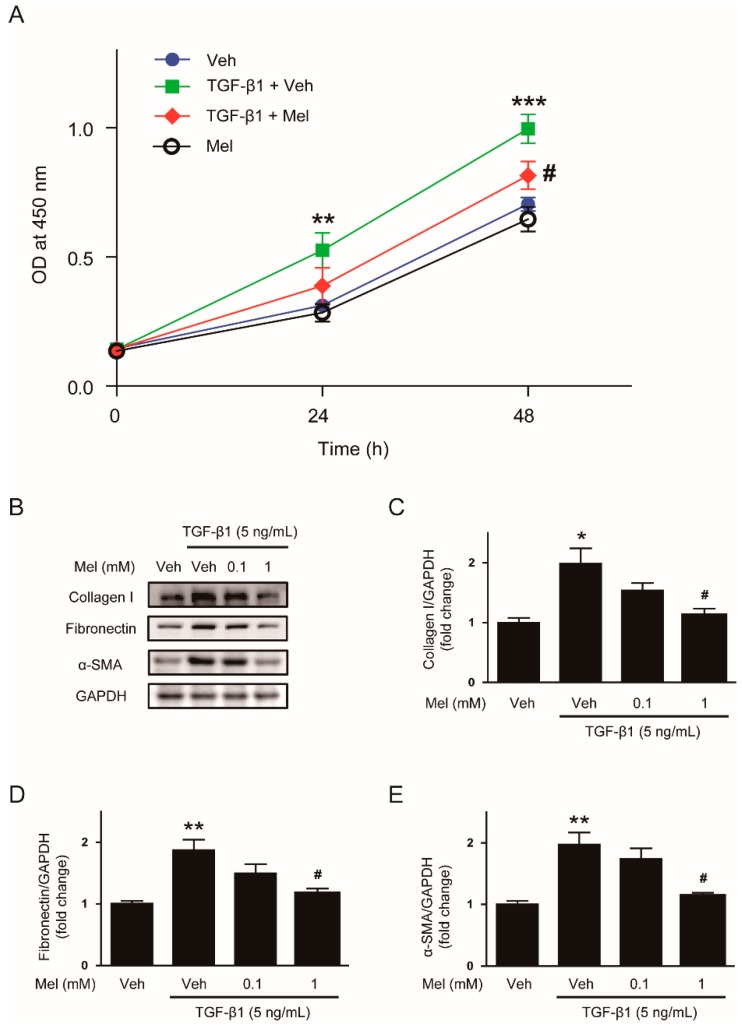
Effects of melatonin on transforming growth factor-β1 (TGF-β1)-stimulated proliferation and activation in renal interstitial fibroblasts. (**A**) NRK-49F cells were treated with TGF-β1 (5 ng/mL) after preincubation with vehicle (Veh) or melatonin (Mel; 1 mM) for 30 min. Cell viability was analyzed using the Cell Counting Kit-8 (CCK-8) assay at 0, 24, and 48 h. The optical density (OD) was measured at 450 nm. (**B**) Western blot analysis for collagen Ⅰ, fibronectin, and α-smooth muscle actin (α-SMA). Cells were treated with TGF-β1 (5 ng/mL) for 24 h after preincubation with Veh or Mel (0.1 mM or 1 mM) for 30 min. The graphs show the results of quantitative analysis of collagen Ⅰ **(C**), fibronectin (**D**), and α-SMA (**E**). * *p* < 0.05, ** *p* < 0.01, and *** *p* < 0.001 vs. Veh-treated cells. ^#^ *p* < 0.05 vs. TGF-β1-treated cells.

Given that proliferation and activation of fibroblasts are key processes for their transdifferentiation to myiofibroblasts, we first investigated the effects of melatonin on TGF-β1-stimulated proliferation of renal interstitial fibroblasts. NRK-49F cells were preincubated with melatonin (1 mM) and then treated with TGF-β1 (5 ng/mL). Cell viability was evaluated using CCK-8 assay at 0, 24, and 48 h. Pretreatment with melatonin significantly suppressed TGF-β1-stimulated proliferation, while melatonin alone did not affect cell proliferation (Figure 1A).

We next examined the effects of the hormone on fibroblast activation invoked by TGF-β1. Treatment with TGF-β1 (5 ng/mL) significantly increased expression of ECM proteins including collagen Ⅰ and fibronectin, and α-SMA when compared with the control (Figure 1B–E). These changes were significantly suppressed by melatonin (1 mM).

### 3.2. Melatonin Suppresses TGF-β1-Induced Smad and Non-Smad Signaling Cascades

**Figure 2:**
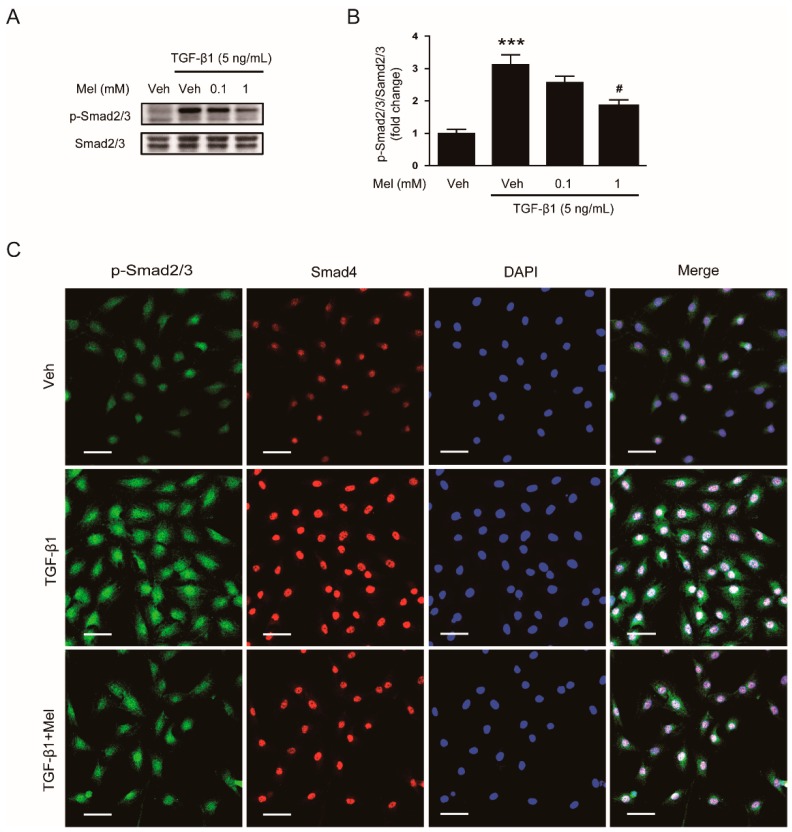
Effects of melatonin on TGF-β1-stimulated activation of Smad signaling pathway in renal interstitial fibroblasts. NRK-49F cells were treated with TGF-β1 (5 ng/mL) for 24 h after preincubation with vehicle (Veh) or melatonin (Mel; 0.1 mM, or 1 mM) for 30 min. (**A**) Western blot analysis for p-Smad2/3 and Smad2/3. (**B**) The graph shows the result of quantitative analysis of p-Smad2/3 (**C**) Representative immunofluorescence staining of p-Smad2/3 (green) and Smad4 (red) in cells treated with Veh, cells treated with TGF-β1 (5 ng/mL), or cells treated with TGF-β1 (5 ng/mL) plus Mel (1mM). Nuclei were stained with 4′,6-diamidino-2-phenylindole (DAPI) (blue). Scale bar: 50 μm. *** *p* < 0.001 vs. Veh-treated cells. ^#^ *p* < 0.05 vs. TGF-β1-treated cells.

In order to explore mechanisms for the inhibitory effects of the hormone on fibroblast-myofibroblast transdifferentiation, we first investigated its effects on TGF-β1/Smad signaling pathway. TGF-β1 induces phosphorylation of Smad2 and Smad3, which form a heteromeric complex with Smad4 [2]. Then, the complex is translocated into the nucleus to regulate expression of fibrosis-related genes. We found that pretreatment with melatonin (1 mM) suppressed TGF-β1-induced phosphorylation of Smad2/3 (Figure 2A,B). Immunofluorescent staining revealed that increased nuclear co-localization of their phosphorylated forms and Smad4 after TGF-β1 treatment was decreased by melatonin (Figure 2C).

**Figure 3:**
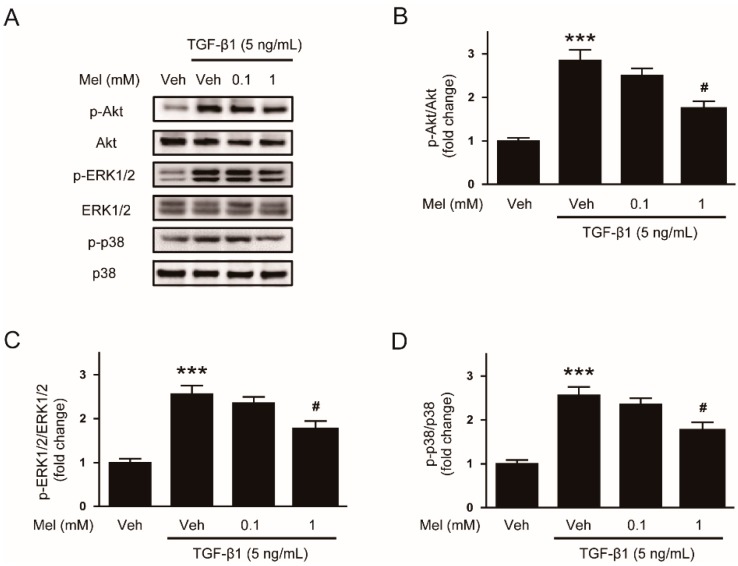
Effects of melatonin on TGF-β1-induced activation of non-Smad signaling pathway in renal interstitial fibroblasts. NRK-49F cells were treated with TGF-β1 (5 ng/mL) for 30 min after preincubation with vehicle (Veh) or melatonin (Mel; 0.1 mM, or 1 mM) for 30 min. (**A**) Western blot analysis for p-Akt, Akt, p-extracellular signal-regulated kinase 1/2 (ERK1/2), ERK1/2, p-p38, and p38. The graphs show the results of quantitative analysis of p-Akt (**B**), p-ERK1/2 (**C**), and p-p38 (**D**). *** *p* < 0.001 vs. Veh-treated cells. ^#^ *p* < 0.05 vs. TGF-β1-treated cells.

In addition, the cytokine can also induce activation of non-Smad signaling pathways such as Akt or MAPK cascades [8]. We observed that phosphorylations of Akt, ERK1/2, and p38 after TGF-β1 treatment were also significantly inhibited by the hormone (1 mM) (Figure 3A–D). Collectively, these findings indicate that melatonin suppresses Smad and non-Smad signaling pathways stimulated by TGF-β1.

### 3.3. Inhibitory Effects of Melatonin on TGF-β1-Induced Proliferation and Activation Is Independent of Its Membrane Receptors

**Figure 4:**
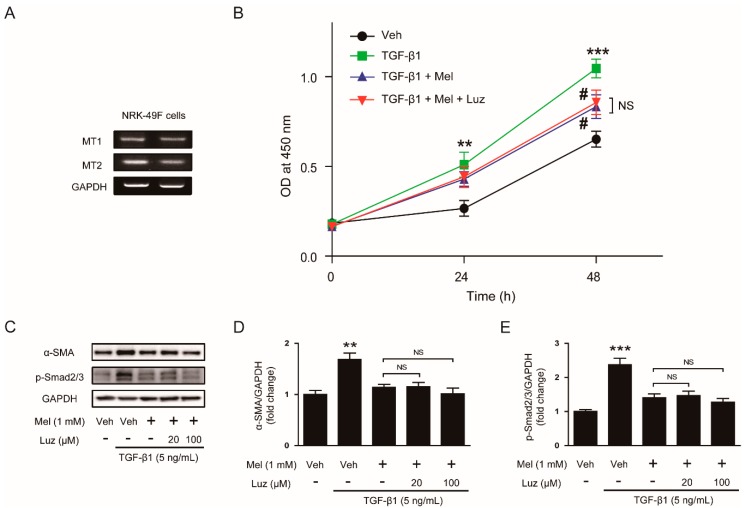
Effects of luzindole on inhibitory action of melatonin on TGF-β1-stimulated proliferation and activation in renal interstitial fibroblasts. (**A**) Reverse transcription-polymerase chain reaction analysis of melatonin receptor type 1A (MT1) and type 1B (MT2) in NRK-49F cells. (**B**) NRK-49F cells were treated with with TGF-β1 (5 ng/mL) after preincubation with vehicle (Veh) or melatonin (Mel; 1mM) in the presence or absence of luzindole (Luz; 100 μM) for 30 min. Cell viability was assessed using CCK-8 assay at 0, 24, and 48 h. The OD was measured at 450 nm. (**C**) Western blot analysis for α-SMA and p-Smad2/3. Cells were treated with TGF-β1 (5 ng/mL) for 24 h after preincubation with Veh or Mel (1 mM) in the presence or absence of luzindole (Luz; 20 μM or 100 μM) for 30 min. The graphs show the results of quantitative analysis of α-SMA (**D**) and p-Smad2/3 (**E**). ** *p* < 0.01 and *** *p* < 0.001 vs. Veh-treated cells. ^#^ *p* < 0.05 vs. TGF-β1-treated cells. NS: not significant.

It has been shown that melatonin displays multiple actions through its membrane receptor-dependent and -independent mechanisms [10]. To date, two subtypes of melatonin membrane receptors, MT1 and MT2, have been identified. To evaluate whether the inhibitory effects of the hormone on proliferation and activation invoked by TGF-β1 is dependent on its receptors, we evaluated the effects of luzindole, an antagonist of melatonin receptors, on the action of melatonin. We first confirmed the presence of MT1 and MT2 in NRK-49F cells using RT-PCR (Figure 4A). Treatment with luzindole at a routinely used concentration (100 μM) [18,19] did not affect the inhibitory effects of melatonin on TGF-β1-stimulated proliferation (Figure 4B). Additionally, increased levels of α-SMA and phosphorylated Smad2/3 after TGF-β1 treatment were not significantly modified by the compound (Figure 4C–E), suggesting that the receptors are dispensable for the suppressive effects of the hormone on TGF-β1-stimulated fibroblast-myofibroblast transdifferentiation.

**Figure 5:**
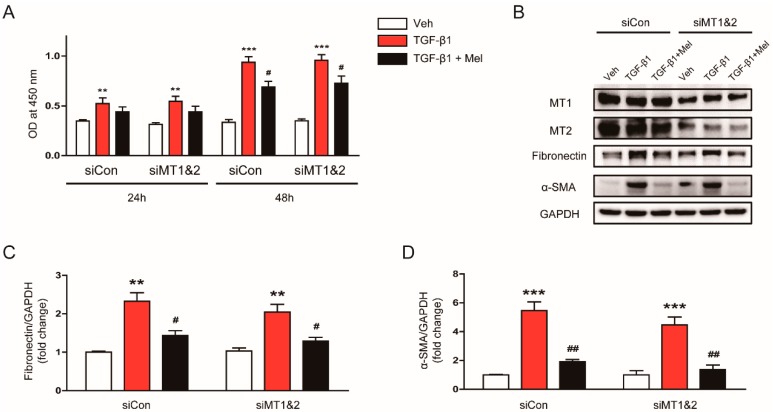
Effects of siRNA-mediated knockdown of melatonin receptors on inhibitory effects of melatonin on TGF-β1-induced proliferation and activation in renal interstitial fibroblasts. NRK-49F cells were treated with negative control siRNA (siCon) or siRNAs targeting MT1 and MT2 (siMT1&B). After 24 h, the cells were pretreated with vehicle (Veh) or melatonin (Mel; 1 mM) and then incubated with TGF-β1 (5 ng/mL) for 24 or 48 h. (**A**) Cell viability was assessed using CCK-8 assay at 24 and 48 h. The OD was measured at 450 nm. (**B**) Western blot analysis for MT1, MT2, fibronectin, and α-SMA. Cells were incubated with TGF-β1 (5 ng/mL) for 24 h after pretreatment with Veh or Mel (1 mM). The graphs show the results of quantitative analysis of fibronectin (**C**) and α-SMA (**D**). ** *p* < 0.01 and *** *p* < 0.001 vs. Veh-treated cells. ^#^ *p* < 0.05 and ^##^ *p* < 0.01 vs. TGF-β1-treated cells.

To more clearly demonstrate that the inhibitory effects of melatonin on fibroblast-myofibroblast transdifferentiation is independent of its receptors, we next examined the effects of genetic inhibition of MT1 and MT2 using siRNA on the action of the hormone. We found that knockdown of melatonin receptors (MT1 and MT2) using siRNA did not significantly affect the inhibitory effects of the hormone on TGF-β1-induced proliferation (Figure 5A) and expression of fibronectin and α-SMA (Figure 5B–D). Altogether, these results suggest that the inhibitory action of melatonin on TGF-β1-stimulated proliferation and activation is independent of its receptors.

### 3.4. Inhibitory Effects of Melatonin on TGF-β1-Induced Proliferation and Activation Is attributed to the Inhibition of ROS-Mediated Mechanisms

**Figure 6:**
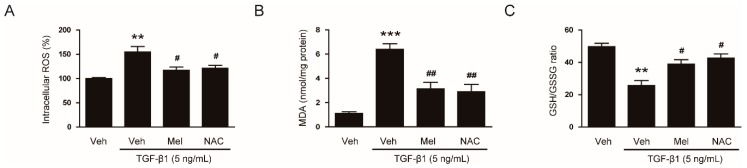
Effects of melatonin or N-acetylcysteine (NAC) on TGF-β1-stimulated generation of reactive oxygen species (ROS) and alterations in redox status. NRK-49F cells were pretreated with vehicle (Veh), melatonin (Mel; 1 mM), or NAC (10 mM) and then incubated with TGF-β1 (5 ng/mL) for 24 h. (**A**) Intracellular ROS levels. (**B**) Intracellular malondialdehyde (MDA) levels. (**C**) The reduced glutathione/oxidized glutathione ratio (GSH/GSSG). ** *p* < 0.01 and *** *p* < 0.001 vs. Veh-treated cells. ^#^ *p* < 0.05 and ^##^ *p* < 0.01 vs. TGF-β1-treated cells.

Melatonin can act as a direct scavenger of ROS. Accumulating evidence suggests that the antioxidant property of melatonin is closely associated with its membrane receptor-independent mechanisms [10]. Thus, we next aimed to explore whether inhibitory action of the hormone on TGF-β1-stimulated fibroblast-myofibroblast transdifferentiation is attributed to the deactivation of ROS-mediated mechanisms. We first investigated the effects of melatonin on ROS generation in NRK-49F cells stimulated by TGF-β1. As expected, treatment with TGF-β1 elevated intracellular levels of ROS and MDA (Figure 6A,B). These effects were significantly attenuated by pretreatment with melatonin or NAC. Decreased GSH/GSSG ratio after TGF-β1 treatment was also significantly reversed by the hormone or NAC (Figure 6C). Altogether, these results suggest that melatonin effectively reduces ROS generation and lipid peroxidation, and changes intracellular redox status in TGF-β1-treated renal interstitial fibroblasts.

**Figure 7:**
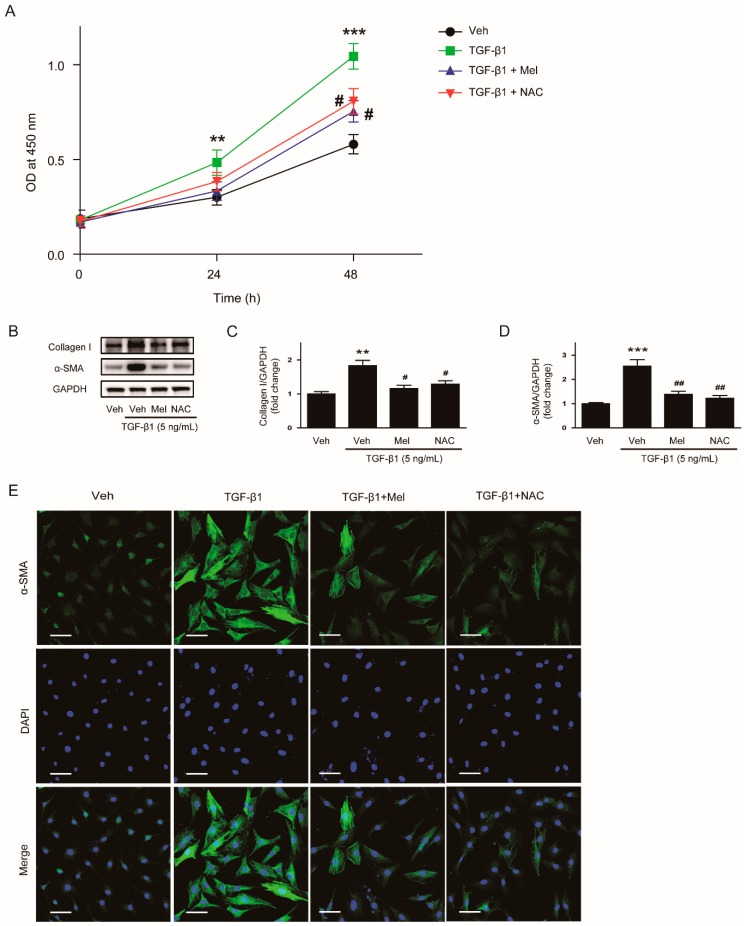
Effects of melatonin or NAC on TGF-β1-stimulated proliferation and activation in renal interstitial fibroblasts. (**A**) NRK-49F cells were treated with TGF-β1 (5 ng/mL) after preincubation with vehicle (Veh), melatonin (Mel; 1mM), or NAC (10 mM) for 30 min. Cell viability was assessed using CCK-8 assay at 0, 24, and 48 h. The OD was measured at 450 nm. (**B**) Western blot analysis for collagen Ⅰ and α-SMA. Cells were pretreated with Veh, Mel (1 mM), or NAC (10 mM) and then incubated with TGF-β1 (5 ng/mL) for 24 h. The graphs show the results of quantitative analysis of collagen Ⅰ (**C**) and α-SMA (**D**). (**E**) Representative immunofluorescence staining of α-SMA (green) in cells treated with Veh, TGF-β1 (5 ng/mL), TGF-β1 (5 ng/mL) plus Mel (1mM), or TGF-β1 (5 ng/mL) plus NAC (10 mM). Nuclei were counterstained with DAPI (blue). Scale bar: 50 μm. ** *p* < 0.01, and *** *p* < 0.001 vs. Veh-treated cells. ^#^ *p* < 0.05 and ^##^ *p* < 0.01 vs. TGF-β1-treated cells.

We next examined whether NAC can also exert inhibitory effects on proliferation and activation in renal interstitial fibroblasts stimulated with TGF-β1. We observed that TGF-β1-stimulated proliferation was significantly inhibited by NAC or melatonin (Figure 7A). Pretreatment with NAC or melatonin also decreased protein expression of collagen Ⅰ and α-SMA after TGF-β1 treatment (Figure 7B–D). Furthermore, immunofluorescent staining revealed that an elevation in α-SMA expression (Figure 7E) after TGF-β1 treatment was reversed by NAC as well as melatonin.

**Figure 8:**
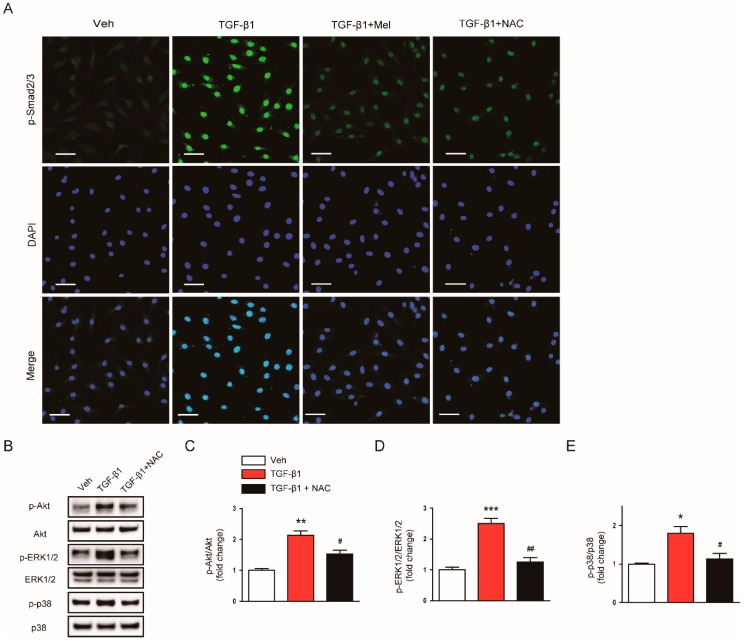
Effects of melatonin or NAC on TGF-β1-stimulated nuclear localization of p-Smad2/3 in renal interstitial fibroblasts. NRK-49F cells were pretreated with vehicle (Veh), melatonin (Mel; 1mM), or NAC (10 mM) and then incubated with TGF-β1 (5 ng/mL) for 24 h. (**A**) The images show representative immunofluorescence staining of p-Smad2/3 (green). Nuclei were counterstained with DAPI (blue). Scale bar: 50 μm. (**B**) Western blot analysis for p-Akt, Akt, p-ERk1/2, ERK1/2, p-p38, and p38. The graphs show the results of quantitative analysis of p-Akt (**C**), p-ERK1/2 (**D**), and p-p38 (**E**). * *p* < 0.05, ** *p* < 0.01, and *** *p* < 0.001 vs. Veh-treated cells. ^#^ *p* < 0.05 and ^##^ *p* < 0.01 vs. TGF-β1-treated cells.

These molecules also suppressed nuclear localization of p-Smad2/3 (Figure 8A) and reversed increased expression of p-Akt, p-ERK1/2, and p-p38 (Figure 8B–E) invoked by TGF-β1. Taken together, these findings indicate that the suppressive effects of melatonin on fibroblast-myofibroblast transdifferentiation invoked by TGF-β1 is presumably attributed to the suppression of ROS-dependent mechanisms.

## 4. Discussion

**Figure 9:**
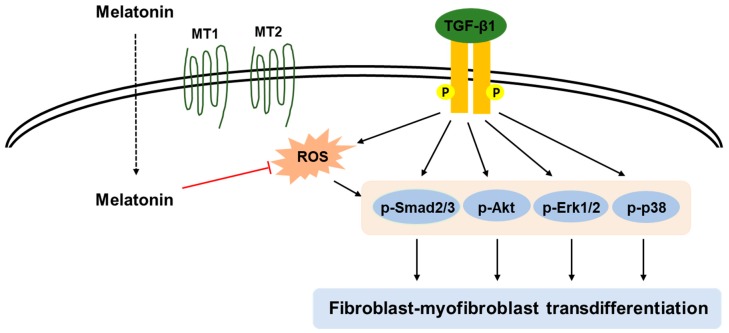
A graphical representation of the mechanism for the inhibitory action of melatonin on TGF-β1-induced transdifferentiation of fibroblasts to myofibroblasts. Melatonin prevents TGF-β1-stimulated proliferation and activation by suppressing Smad and non-Smad signaling cascades (Akt, ERK1/2, and p38) in renal interstitial fibroblasts. These effects of melatonin were attributed to the deactivation of ROS-dependent mechanisms in its membrane receptors (MT1 and MT2)-independent manner.

In this study, we demonstrated that melatonin inhibited TGF-β1-stimulated transdifferentiation of renal interstitial fibroblasts to myofibroblasts. Pretreatment with melatonin effectively suppressed TGF-β1-stimulated proliferation. Increased levels of activation-related markers after TGF-β1 treatment was also inhibited by the hormone. These effects of the hormone were accompanied by suppression of Smad and non-Smad signaling pathways (Akt, ERK1/2, and p38). Additionally, pharmacological ad genetic inhibition of melatonin receptors (MT1 and MT2) did not modify the action of melatonin, indicating that the receptors are not required for the suppressive effects of the hormone on TGF-β1-induced proliferation and activation. Furthermore, we found that the suppressive effects of melatonin on TGF-β1-induced transdifferentiation of fibroblasts to myofibroblasts are presumably attributed to the suppression of ROS-dependent mechanisms. These results provide a novel mechanistic insight into the preventive effects of the pineal hormone on renal fibrosis (Figure 9).

The pineal hormone melatonin plays an essential role in modulating the sleep–wake cycle [9]. Besides, the hormone has been shown to exert preventive and/or therapeutic effects against various diseases [10,11]. The favorable actions of melatonin were primarily attributed to its anti-inflammatory and anti-oxidant activities. Emerging evidence also reveals that the hormone exerts strong anti-fibrotic activities in various organs [12]. It has been shown that melatonin ameliorated cyclosporine A [13]- or carbon tetrachloride [14]-induced renal fibrosis. Additionally, melatonin was found to suppress the fibrotic process invoked by unilateral ureteral obstruction (UUO) in a rodent model of CKD [15]. A recent study also showed that melatonin ameliorated renal fibrosis in animals with diabetic kidney disease [16]. However, molecular mechanisms for the favorable effects of the hormone against renal fibrosis remain unclear. In the present study, we demonstrated that melatonin prevents transdifferentiation of renal interstitial fibroblasts to myofibroblasts invoked by TGF-β1. During the development and progression of fibrosis, myofibroblasts synthesize and secret ECM components including collagen and fibronectin. Given that fibroblast-myofibroblast transdifferentiation is a critical process in the pathophysiology of renal fibrosis [1], these findings provide a novel mechanistic insight into the preventive effects of melatonin against renal fibrosis. Consistent with our findings, a previous study showed that melatonin inhibits TGF-β1-induced epithelial-mesenchymal transition in lung alveolar epithelial cells through suppressing Smad and Wnt/β-catenin signaling pathways [20]. The hormone was also found to suppress fibrotic process in rat kidneys and human renal proximal tubular epithelial cells by inhibiting Smad and MAPK signaling pathways [21]. Additionally, it was recently reported that the anti-fibrotic effects of melatonin against liver fibrosis induced by carbon tetrachloride was associated with its inhibitory action on TGF-β1/Smad signaling cascade [22].

Canonical TGF-β/Smad signaling cascade plays a key role in the regulation of fibroblast-myofibroblast transdifferentiation [2,3]. TGF-β1 induces phosphorylation of Smad2 and Smad3, which form a heteromeric complex with Smad4. Then, the complex is translocated into the nucleus to regulate expression of fibrosis-related genes. In this study, we noted that melatonin suppressed TGF-β1-stimulated phosphorylation of Smad2/3. Furthermore, immunofluorescent staining showed that the hormone significantly attenuated TGF-β1-induced nuclear co-localization of their phosphorylated forms and Smad4. In addition, the cytokine can activate various non-Smad signaling pathways [8]. We also observed that melatonin significantly suppressed an increase in the phosphorylations of Akt, ERK1/2, and p38 after TGF-β1 treatment. Previous studies showed that Akt and ERK1/2 in ligated kidneys were activated in the UUO model [23]. Pharmacological inhibition of Akt or ERK1/2 induces a reduction in levels of myofibroblast markers in the kidneys. In addition, phosphorylation of p38 was also shown to be increased in the UUO model [24,25] and a genetic model of CKD [26]. Treatment with a specific inhibitor of p38 significantly reduced accumulation of interstitial myofibroblasts and renal fibrosis in both models. Collectively, our findings suggest that melatonin dampens TGF-β1-induced fibroblast-myofibroblast transdifferentiation through inhibition of Smad and non-Smad signaling pathways.

Melatonin displays various biological actions through both its receptor-dependent and -independent mechanisms [10]. To date, two subtypes of mammalian membrane receptors, MT1 and MT2, have been identified. Membrane receptor-dependent action of melatonin includes regulation of circadian rhythm and anti-cancer effect [10]. In addition, a number of studies have reported the receptor-independent action of melatonin on various cellular functions despite existence of melatonin receptors [27,28,29,30]. In the present study, luzindole, an antagonist of melatonin receptors, was used to evaluate whether the suppressive effects of the hormone on TGF-β1-stimulated fibroblast-myofibroblast transdifferentiation are dependent on its receptors. We found that the compound did not modify the suppressive effects of melatonin on TGF-β1-induced proliferation and activation of fibroblasts. Furthermore, the effects of melatonin were not significantly affected by siRNA-mediated knockdown of MT1 and MT2, indicating that the receptors are not required for the suppressive effects of the hormone. The membrane receptor-independent actions of melatonin have been known to be related to its ROS scavenging property [10]. Because of its high lipophilicity, melatonin can easily enter into the cytosol through passing the plasma membrane. In cytosol, the hormone can directly scavenge ROS. In the present study, we noted an elevation of intracellular ROS levels after TGF-β1 treatment. This observation is consistent with the results of previous reports [31,32,33]. As expected, melatonin significantly attenuated ROS production and changed intracellular redox status in renal interstitial fibroblasts stimulated by TGF-β1, as evidenced by decreased intracellular levels of ROS and MDA and increased GSH/GSSG ratio. We also found that the antioxidant NAC also exerted inhibitory effects on TGF-β1-induced proliferation and activation. Immunofluorescent staining clearly revealed that an elevation in α-SMA expression and nuclear localization of p-Smad2/3 after TGF-β1 treatment were reversed by NAC as well as melatonin. Pretreatment with NAS also suppressed phosphorylations of Akt, ERK1/2, and p38 after TGF-β1 treatment. Taken together, these findings indicate that the suppressive action of melatonin on fibroblast-myofibroblast transdifferentiation invoked by TGF-β1 is presumably attributed to the suppression of ROS-mediated mechanisms in its receptor-independent manner.

ROS can act as an intracellular second messenger during cell differentiation [34]. Indeed, accumulating evidence suggests that nicotinamide adenine dinucleotide phosphate (NADPH) oxidase-generated ROS is critically involved in fibroblast-myofibroblast transdifferentiation and progression of kidney fibrosis [31,35]. TGF-β1-stimulated conversion of fibroblasts into myofibroblasts in other organs including heart [36], lung [37], intestine [38], and skin [39] was also mediated by ROS derived from NADPH. Thus, targeting of NADPH oxidase is considered as a potential preventive and therapeutic strategy against fibrotic diseases. Altogether, our findings suggest that the ROS scavenging property of melatonin mainly contributes to its suppressive effects on TGF-β1-stimulated fibroblast-myofibroblast transdifferentiation.

## 5. Conclusions

These results demonstrate that the pineal hormone melatonin prevents TGF-β1-stimulated transdifferentiation of fibroblasts to myofibroblasts by suppressing Smad and non-Smad signaling cascades. These effects of melatonin were attributed to the deactivation of ROS-mediated mechanisms in its receptor-independent manner. These results strengthen the idea that melatonin may be a promising preventive option against fibrotic diseases.
